# Optical Frequency Domain Reflectometry Based on Multilayer Perceptron

**DOI:** 10.3390/s23063165

**Published:** 2023-03-16

**Authors:** Guolu Yin, Zhaohao Zhu, Min Liu, Yu Wang, Kaijun Liu, Kuanglu Yu, Tao Zhu

**Affiliations:** 1 Key Laboratory of Optoelectronic Technology & Systems (Ministry of Education), Chongqing University, Chongqing 400044, China; 2 State Key Laboratory of Coal Mine Disaster Dynamics and Control, Chongqing University, Chongqing 400044, China; 3 School of Microelectronics & Communication Engineering, Chongqing University, Chongqing 400044, China; 4 Institute of Information Science, School of Computer and Information Technology, Beijing Jiaotong University, Beijing 100044, China; 5 Beijing Key Laboratory of Advanced Information Science and Network Technology, Beijing 100044, China

**Keywords:** optical frequency domain reflectometry, machine learning, strain measurement, multilayer perceptron

## Abstract

We proposed an optical frequency domain reflectometry based on a multilayer perceptron. A classification multilayer perceptron was applied to train and grasp the fingerprint features of Rayleigh scattering spectrum in the optical fiber. The training set was constructed by moving the reference spectrum and adding the supplementary spectrum. Strain measurement was employed to verify the feasibility of the method. Compared with the traditional cross-correlation algorithm, the multilayer perceptron achieves a larger measurement range, better measurement accuracy, and is less time-consuming. To our knowledge, this is the first time that machine learning has been introduced into an optical frequency domain reflectometry system. Such thoughts and results would bring new knowledge and optimization to the optical frequency domain reflectometer system.

## 1. Introduction

Since Wc Eickhoff et al. proposed the optical frequency domain reflectometry (OFDR) sensing system in 1981 [1], due to its advantage of high spatial resolution, it is now widely used in distributed measurement fields such as temperature measurement [2,3,4,5], strain measured [6,7,8,9], and shape sensing [10,11,12,13,14,15]. The cross-correlation algorithm (CCA) is a common data processing algorithm for OFDR systems, which calculates the wavelength shift value by comparing spectrum fingerprint peaks between the reference signal and the measured signal. However, the fingerprint peaks in the spectrum of the reference and the measured signal are not identical. Compared with the reference signal, the spectrum of the measured signal will have random multiple peaks and pseudopeaks affecting the precision of the CCA. Therefore, it is necessary to propose a new method to solve this problem of CCA.

Machine learning could learn signal features and regularity from large amounts of data and process them. In the field of distributed optical fiber sensing, several machine learning methods have been applied to improve the sensing performance in Brillouin optical time domain analysis (BOTDA) [16,17,18], Brillouin optical time domain reflectometer (BOTDR) [19,20,21], and phase-sensitive optical time domain reflectometer (Φ-OTDR) [22,23,24,25]. Yang G. et al. proposed a convolutional neural network (CNN) model that consists of a one-dimensional denoising convolutional self-encoder and a one-dimensional residual attention network module; this model could extract both temperature and strain in a BOTDA system with better noise immunity and robustness under the conditions of wider temperature and strain ranges [26]. Compared with the conventional equation-solving method, the CNN method improved temperature and strain identification accuracy to 196 times and the processing speed to 146 times. Zheng H. et al. proposed a Fastdvdnet algorithm to process vibration signals from fast BOTDA systems with a spatial resolution of 2 m and a length of 10 km [27]. Since Fastdvdnet used GPU for acceleration during data processing, it took only 0.038 s to process 100 × 21,800 data, which provided a research idea for realizing fast vibration sensing of long-distance BOTDA. Cao Z. et al. used a neural network to improve the localization accuracy of BOTDA system [28]. Compared with the Lorentz fitting algorithm, the BP neural network was less affected by noise, the time complexity was only 1/12 of the Lorentz fitting algorithm, and the localization accuracy was 79.4% higher. Wu H. et al. demonstrated that, in the BOTDA system, a support vector machine improved the temperature measurement accuracy by 30%, and the measurement speed was 80 times faster than the conventional algorithm [29]. Madaschi A. et al. proposed that the temperature data of the BOTDA system can be enhanced by using a multiple neural network and a single neural network [30]. Compared with the three standard fitting algorithms, the multiple neural network provides the best measurement with a 7.8% gain while significantly reducing the measurement time. Hu Y. et al. used a generative adversarial network for the BOTDA system to improve the performance of system data processing [31]. Compared with the direct measurement method, the generative adversarial network reduces the measurement volume by 75%, increases the sensing speed four times, and improves the spatial resolution significantly. Chen B. et al. used a wavelet convolutional neural network to reduce the temperature measurement time of BOTDR by three orders of magnitude [32]. The wavelet convolutional neural network takes only 0.54 s to process 18,000 temperature Brillouin gain spectra, which were collected under 18 km of single-mode-fiber conditions. Wu H. et al. used bidirectional long–short-term memory based on one-dimensional convolution neural networks to improve the vibration event recognition rate of a distributed acoustic sensor system to 98.6% [33]. Li S. et al. proposed a CNN image denoising model that can effectively reduce the noise in the phi-OTDR system, reduce the denoising time, and improve the denoising effect [34]. This model was validated on a 41 km sensing fiber. Compared with wavelet transform and empirical mode decomposition, CNN achieved an average 20 dB SNR improvement in vibration signals of different amplitudes. Tian M. et al. applied an attention-based temporal convolutional network combined with spatial attention and a bidirectional long–short-term memory network model for the classification task of phi-OTDR, focusing on extracting the relational features of time domain signals and spatial sequences, reducing the bidirectional propagation of interference in the space domain signals, and improving the classification accuracy to 93.4% with a zero false alarm rate [35]. Shi Y. et al. designed a less sample learning classification method based on time series migration and recurrent generative adversarial networks, which solved the problem of insufficient training samples due to the difficulty of vibration signal collection, making the neural network unable to identify accurately [36]. The algorithm demonstrated that even if the training set had only two small categories with only two samples per category, the classification task of five vibrational events could achieve an average accuracy of 90.84%, and the accuracy of the small category classification could reach 79.28%. For OFDR systems, Rayleigh backscattering has specific fingerprint information in the frequency domain, which is beneficial for feature extraction in the machine learning method. The machine learning method would be promising in obtaining the fingerprint features of Rayleigh scattering spectra. 

In this paper, a multilayer perceptron (MLP) was used to obtain features of the Rayleigh scattering spectrum in the OFDR system. To our knowledge, this is the first time that a machine learning method has been introduced into the OFDR system. The training spectra were used as the training set to train the MLP, and the feasibility of the principle was verified by applying the strain measurement. The comparative experiments of CCA and the machine learning method confirmed the reliability of the measurement results, and our proposed method has a larger measurement range, better measurement accuracy, and it is less time-consuming.

## 2. Theory

The OFDR system is a distributed fiber optic sensing system based on backward Rayleigh scattering; the basic principle is based on the coherent detection technology of an optical-frequency-modulated continuous wave. When applying strain to the test fiber, the phase of the measurement light in the test fiber will change, causing the fingerprint peaks in the Rayleigh scattering spectra to shift. CCA calculates wavelength shift by demodulating the distance of the fingerprint peak shift to achieve distributed measurement of strain.

The steps for CCA to calculate wavelength shift are as follows. Firstly, we acquire the reference signal before applying strain and the measurement signal after applying strain in the time domain, and then the two time domain signals are converted to frequency domain signals by using the Fourier transform. In addition, there is a linear relationship between the frequency domain and the distance domain, so the two signals can be converted from the frequency domain to the distance domain by using the fast Fourier transform. Then, the moving window is intercepted at the same position as the two sets of distance domain signals, and the length of the moving window can be freely set according to the sensing requirements. It should be noted that in the OFDR system, the length of the moving window will affect the sensing performance, such as data size and spatial resolution, so it is necessary to set the appropriate window length. For two sets of distance domain signals, a short-time Fourier transform is used for the moving window to obtain the Rayleigh scattering spectra at the window positions. There are fingerprint peaks in the Rayleigh scattering spectra of the reference and measurement signals; the CCA can demodulate the distance between the fingerprint peaks of the two sets of signals and then calculate the wavelength shift at the position of the moving window. Finally, the position of the moving window is changed, and the above steps are repeated to obtain the wavelength shift values at all positions on the test fiber, realizing the distributed strain measurement.

**Figure 1:**
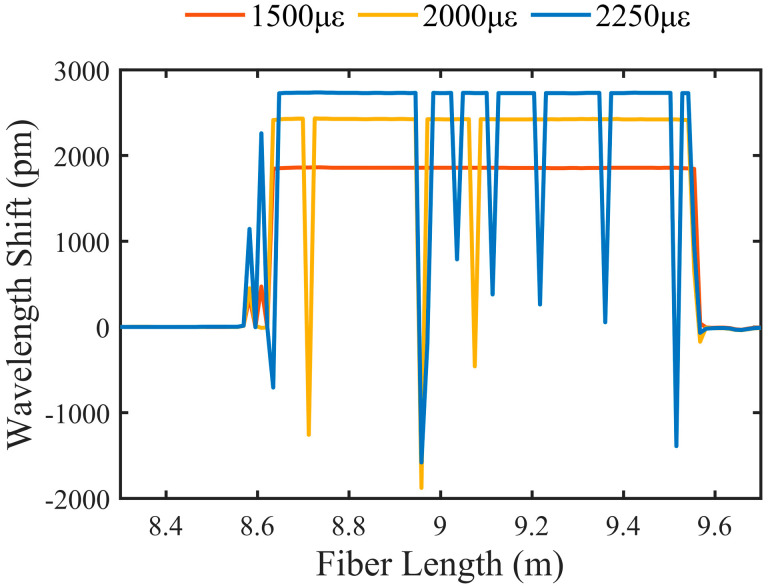
Wavelength shift calculated by cross−correlation algorithm at strains of 1500, 2000, and 2250 με.

Figure 1 shows the wavelength shifts of Rayleigh scattering spectra by using the CCA. We acquired the reference and measurement Rayleigh scattering spectra before and after strain change, respectively. The fingerprint peaks in the spectra move linearly with strain. The CCA is used to calculate moving distance, and hence, the wavelength shift corresponding to the strain is obtained. The wavelength shift can be correctly obtained at a strain of 1500 με, while error points appear at strains of 2000 and 2250 με. The Rayleigh scattering spectra move linearly with the strain, and new spectral components are generated in the spectral range, reducing the overlap between the reference and measurement spectra. According to experimental results, error points in the wavelength shifts are found when the overlap coefficient is reduced to around 50%. The generation of new spectral components and a reduction in the overlap would cause multipeaks and pseudopeaks of cross-correlation, and hence, errors are found in the wavelength shift when the strain exceeds an upper limit. In other words, the limitation of the CCA is that the random multipeaks and pseudopeaks make many errors in the strain measurement, and this limitation is especially obvious in the large strain measurement.

**Figure 2:**
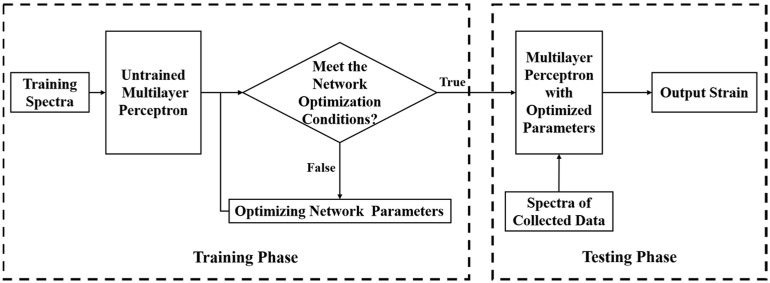
Schematic diagram of training phase and testing phase of multilayer perceptron.

To overcome the limitation of the CCA, a machine learning method is introduced to grasp the characteristics of the Rayleigh scattering spectrum, making the algorithm work even when the overlap between reference and measurement spectra is largely reduced. We chose an MLP model based on the backpropagation algorithm to process the strain data. MLP is a traditional, fully connected neural network that consists of an input layer, hidden layers, and output layer, in which the number of hidden layers can be set according to research needs. MLP currently plays an important role in dealing with regression and classification problems, and it has been successfully applied to image processing [37], semantic processing [38], and financial analysis [39]. The schematic diagram of MLP used in this manuscript to process OFDR data is shown in Figure 2. We first established an initial MLP with random initial values and then used the training spectra as the training set of the initial neural network to train the network and optimize the network parameters. The principle of the composition of the training spectra will be given in the latter part of this section. The MLP is evaluated by the minimum target training error, maximum number of iterations, minimum performance gradient, and other parameters to determine whether the network is optimized. Finally, the experimentally collected spectra are used as the testing set to evaluate the trained neural network’s performance.

A critical phase is the construction of the training and testing sets for the machine learning method. In the traditional machine learning method, the training set and testing set often need to collect a large amount of data and divide the data into two parts. The part with a greater quantity is used as the training set to train the network, and the part with less quantity is used as the testing set to test the network. However, if the same approach is taken for training and testing the neural network in an optic fiber sensing system, it will undoubtedly consume substantial experimental resources and acquisition time, and the model may not be applicable after changing the measurement environment.

**Figure 3:**
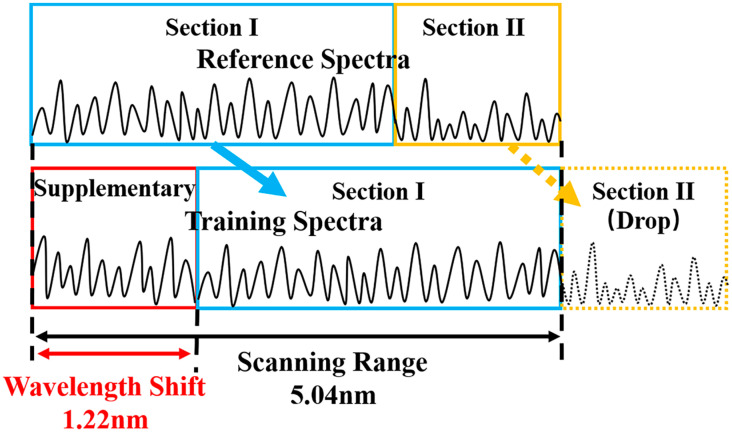
Generation of training set samples in the multilayer perceptron.

To overcome the problem above, we constructed the training set by moving the reference spectra and adding supplementary spectra. Firstly, the reference spectra of the fiber under test are obtained by experiment. Secondly, the reference spectra are moved with a certain shift, and the shift amount is determined by the strain sensitivity of the FUT. Here, the strain sensitivity is around 1.22 pm/με. Finally, new spectral components are added to the training spectra to supplement the moving part of the reference spectra. To describe the generation process of training spectra more intuitively, we take the strain of 1000 με, for example, in Figure 3. The reference spectra are experimentally obtained in a scanning wavelength range of 5.04 nm. The reference spectra are moved 1.22 nm toward the long wavelength direction, and the supplementary spectrum is added in the short-wavelength direction. The blue and yellow denote the part of the reference spectra, after moving the reference spectra, the yellow part is dropped and the red part of the supplementary spectra is added. The supplementary spectrum is generated by the spectrum model of Rayleigh backscattering light [40].

(1) $$b\left(\lambda \right)=\sum_{k}c_{k}\exp(\frac{-4j\pi z_{k}}{\lambda }),$$

where *b*(*λ*) is the value of the supplementary spectrum, *z_k_* and *c_k_* are positions and reflection coefficients of the scattering centers, and *λ* is the wavelength.

**Figure 4:**
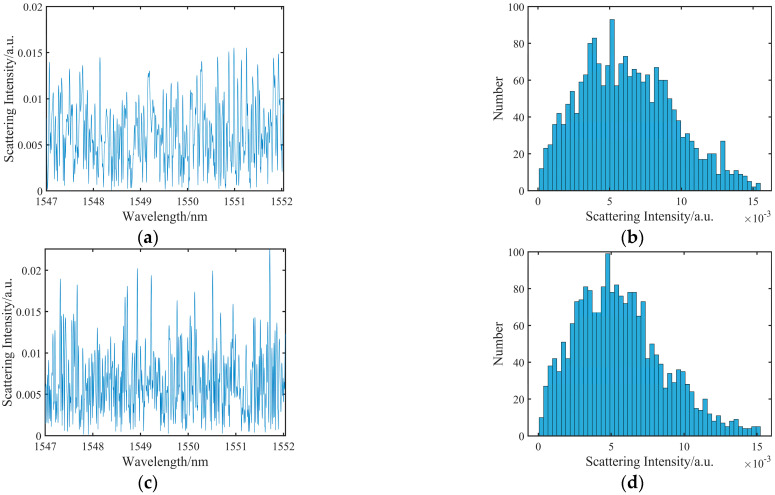
(**a**)Experimental spectrum and (**b**) its Rayleigh scattering distribution; (**c**) training Rayleigh scattering spectrum and (**d**) its Rayleigh scattering distribution.

Following is an experimental description of the supplementary spectra constructed by using the spectra model of Rayleigh backscattering light. We take the data of 1000 με as an example; Figure 4a,b shows the spectra and their distribution obtained by experimental collection. Figure 4c,d shows the training spectra and their distribution, in which the training spectra are composed of the reference spectra and the supplementary spectra. The reference spectra are 0 με data acquired by the OFDR system. The supplementary spectra are composed of the spectra model of Rayleigh backscattering light based on a theoretical wavelength shift of 1000 με. Training spectra are obtained by stitching the supplementary spectra and a part of the reference spectra. As can be observed from the figures, the collected and training spectra basically obey the Rayleigh distribution, which is consistent with the theoretical basis of OFDR. At the same time, in this manuscript, we determine whether the training spectra satisfy the fundamentals of the OFDR system by fitting the relationship between the average free spectra range (FSR) and spatial resolution. The average FSR refers to the average distance between adjacent spectral peaks of Rayleigh scattering spectra, and based on the principle of Michelson interference, the expression of FSR is

(2) $$FSR=\frac{\lambda_{c}^{2}}{2nNz},$$

where *λ_c_* is the central wavelength, *n* is the refractive index of the fiber, *z* is the interval of adjacent points in the distance domain, and *N* is the length of the moving window. Spatial resolution is an important performance indicator of the OFDR sensing system, which indicates that the sensing system can resolve the closest distance between two measurement points on the test fiber. It can be expressed as

**Figure 5:**
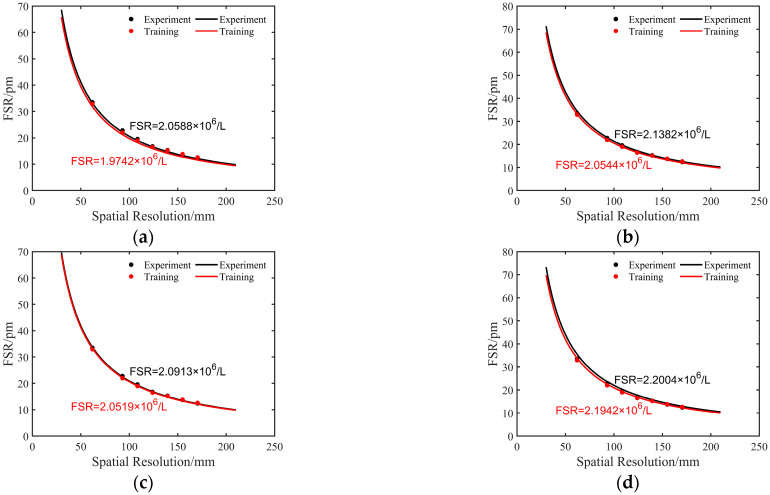
Comparison of free spectra range between experimental and training spectra at strain of (**a**) 100, (**b**) 1000, (**c**) 2000, and (**d**) 2900 με: L, spatial resolution.

(3) L=Nz,

where *L* is spatial resolution. We change the spatial resolution by changing the moving window length *N*. When *N* is increased, the maximum beat frequency at the moving window position increases, the spectral peaks of Rayleigh scattering spectra become denser, and the average distance between spectral peaks becomes smaller. Therefore, the FSR is inversely related to the spatial resolution in the OFDR system. We compared the average FSR of experimental collected spectra and training spectra by using data of 100, 1000, 2000, 2900 με, and the results are shown in Figure 5. The FSR fitted curves of the experimental collected spectra and the training spectra have basically no difference—the two curves are very close, and both are inversely proportional to the spatial resolution, conforming to the basic principle of OFDR. Combined with the above findings, the training spectra have a strong correlation with the experimental collected spectra, in which the training spectra are composed of supplementary spectra based on the spectra model of Rayleigh backscattering light, so it is feasible to construct supplementary spectra by using the spectra model of Rayleigh backscattering light.

## 3. Experiment Results and Discussion

**Figure 6:**
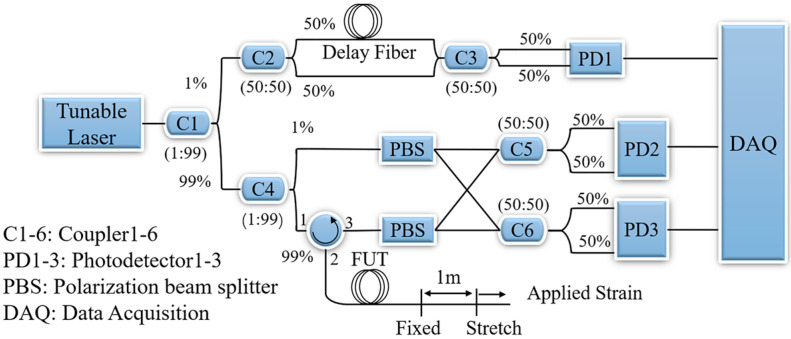
Schematic of the optical frequency domain reflectometry system.

Figure 6 illustrates the OFDR system in our experiment. The optical light from a tunable laser (Phoenix 1202, Luna) is split into two parts and connected with two interferometers. The auxiliary interferometer (upper branch) is an unbalanced Mach Zehnder interferometer with a 202.2 m delay fiber. The main (lower branch) interferometer is used to collect the Rayleigh scattering signal. A polarization diversity system with two polarization beam splitters is employed to weaken polarization fading. The signals from the auxiliary interferometer and main interferometer are collected by a four-channel data acquisition card. The auxiliary interferometer is designed to measure the instantaneous frequency of the laser and then compensate for the nonlinear sweep effect of the laser. The laser scanning speed is 40 nm/s, and the data acquisition time is 0.126 s, giving a laser sweep range of 5.04 nm. The length of the fiber under test (FUT) is 10 m. The optical fiber at 8.6 and 9.6 m was fixed on two linear translation stages. The strain is introduced by moving one of the linear translation stages, and the strain value is obtained by calculating the relative tensile length.

Our dataset of the MLP model includes 17 classes of strains using the OFDR system described above, i.e., 60, 70, 80, 90, 100, 200, 300, 400, 500, 1000, 1500, 2000, 2500, 2600, 2700, 2800, and 2900 με. For a certain strain, 160 training data were generated according to the training spectra generation steps above, and 40 test data were collected experimentally.

Before testing the sensing performance based on MLP, we first determined the network parameters. The training function, activation function, and the number of network layers are optimized in succession. We used classification accuracy as the objective of parameter optimization. We ran the neural network ten times continuously and counted the accuracy. The training function is to modify the global weights and thresholds by minimizing the error, which is applied in the backpropagation process. The activation function is applied to the calculation of weights and thresholds of neurons. Its main role is to complete the nonlinear transformation and normalization of the data. The application of activation functions greatly increases the ability of neural networks to handle nonlinear data and prevents data expansion.

**Figure 7:**
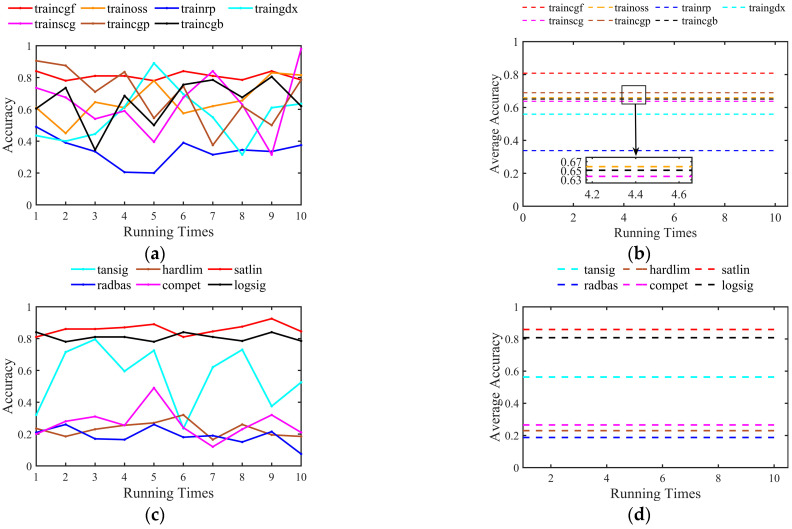
Comparison of the accuracy with different (**a**,**b**) training functions and (**c**,**d**) activation functions.

As shown in Figure 7a,b, seven training functions are tested, and traincgf was chosen as the training function of MLP, and its average accuracy is 80.8%. Figure 7c, d shows the effect of different activation functions on the accuracy of the MLP. After comparison, we chose the satlin function as the activation function of MLP, with an average accuracy of 85.9%.

**Figure 8:**
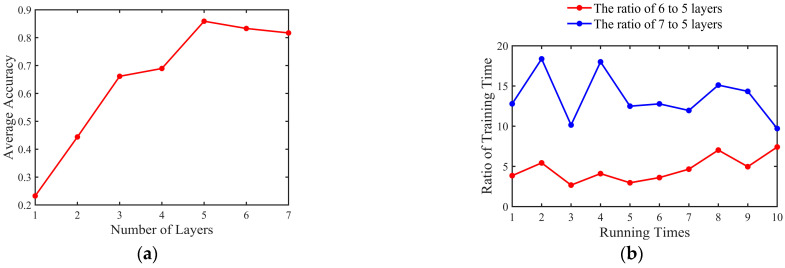
(**a**) Accuracy of multilayer perceptron with different numbers of layers; (**b**) ratio of training time of 7-hidden-layer and 6-hidden-layer to 5-hidden-layer network, respectively.

**Figure 9:**
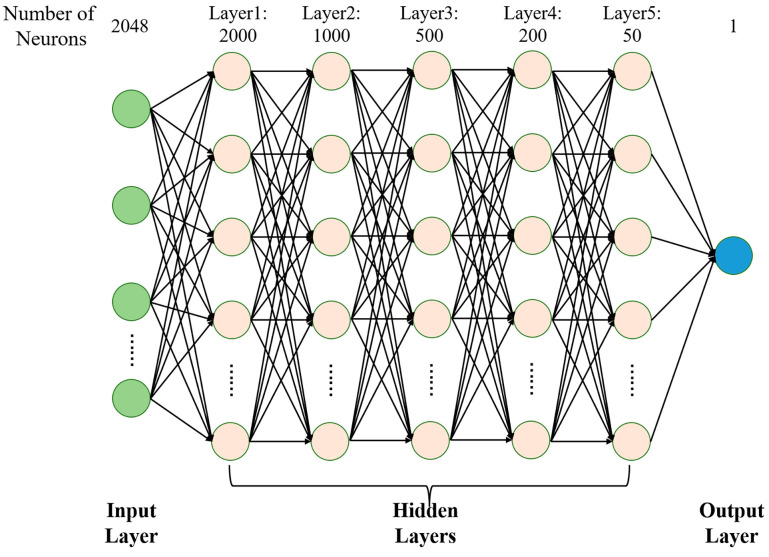
The structure of the proposed multilayer perceptron.

We used traincgf as the training function and satlin as the activation function and ran the network ten times consecutively to obtain the effect of different hidden layers of the neural network on the accuracy rate, as shown in Figure 8. Figure 8a shows the accuracy rates after running ten times, and we can observe that the average accuracy of the 5-hidden-layer, 6-hidden-layer, and 7-hidden-layer networks is relatively close. Therefore, we compared the training time of these three networks with different layers, as shown in Figure 8b. Training time is the time used by the MLP model during the training phase. We found that the training time of the 5-hidden-layer network was the shortest. In the final classification model, we set the learning rate to 0.005, the training function to traincgf, the activation function to satlin, the number of hidden layer layers to five, and the loss function to mean square error. Figure 9 shows the MLP with the five-hidden-layer network structure. Green circles represent input layer neurons, light yellow circles represent hidden layer neurons, and blue circle represents output layer neuron. The input layer of the MLP is the OFDR spectra with a dimension of 2048. After the input layer, there are five hidden layers, and the number of neurons in each hidden layer is 2000, 1000, 500, 200, and 50, respectively. Once the data are processed by the hidden layer, they are passed to the output layer of the MLP to output the result.

We compare the accuracy of the MLP and the CCA by the mean absolute error (*MAE*),

(4) $$MAE=\frac{1}{m}\sum_{i=1}^{m}\left|y_{i}-y_{label}\right|,$$

where *m* is the test time for each strain, *y_i_* is the result obtained by the algorithm calculation, and *y_label_* is the experimental preset value.

**Figure 10:**
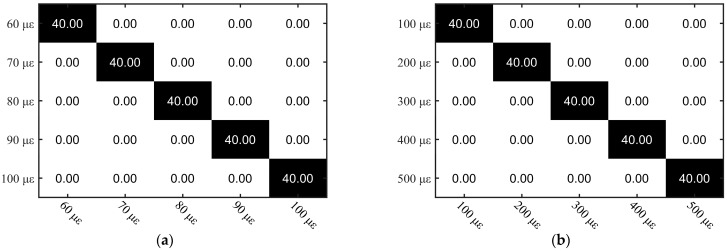
Confusion matrix of multilayer perceptron at strain from (**a**) 60 to 100 με and (**b**) 100 to 500 με.

**Figure 11:**
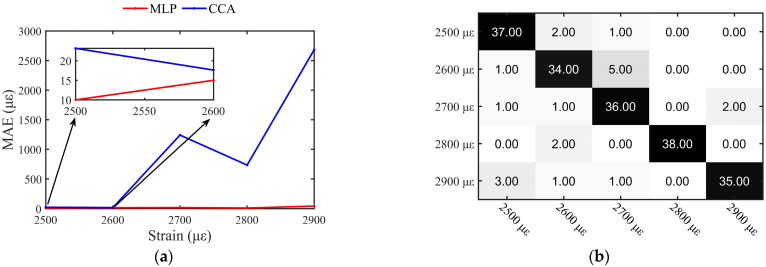
(**a**) Mean absolute error of multilayer perceptron and cross-correlation algorithm at strain from 2500 to 2900 με; (**b**) confusion matrix of multilayer perceptron.

Figure 10 shows the confusion matrix of the MLP for small strain measurements from 60 to 100 με and 100 to 500 με. In the confusion matrix, the color shades represent the amount of data. The smaller the number, the lighter the color, and the larger the number, the darker the color. In the confusion matrix of this paper, the quantity is white when it is 0, gray when it is less, and black when it is more. From the measurement results, the MLP for small strain measurement works perfectly with an accuracy of 100%, and measurement precision can reach 10με with a small error. Figure 11 shows the results of the MLP and the CCA for large strain measurements from 2500 to 2900 με. In Figure 11a, we compared the MAE of MLP to CCA. It is found that the MAE of MLP is smaller than that of the CCA. Once the strain exceeds 2600 με, it is worth pointing out that the CCA totally failed to determine the strain while the MLP still works well. For the strain from 2500 to 2900 με, the corresponding wavelength shift is around 3.08 to 3.57 nm, which means that the overlap between the reference and measurement Rayleigh scattering spectrum is only 29% to 39%. The correlation between spectra is severely reduced, which is the reason why the CCA fails. In contrast, MLP can still find recognition features from the remaining spectra, thus obtaining 90% classification accuracy, as shown in the confusion matrix in Figure 11b.

The algorithm was running on a Windows 10 computer with a 64-bit CPU intel(R) Core (TM) i7-11800H, where the CPU is running at 2.30 GHz. Table 1 compares the running time of the MLP and the CCA. Both methods computed 400 sets of test data, and each method was performed four times. The comparison clearly shows that the CCA costs an average time of 18.8 s, while the MLP method only costs an average time of 0.446 s, saving more than 40 times. Therefore, the MLP demonstrates amazing speed in processing the OFDR data.

## 4. Conclusions

In this paper, for the first time, we introduced machine learning into the OFDR system, and the MLP was used to train the fingerprint information of the Rayleigh backscattering spectra in single-mode fiber. The training spectrum was generated by moving the reference spectra and adding supplementary spectra. In total, we generated 17 kinds of training sets with different strains, and each strain contains 160 training sets. The training function, activation function, and the number of network layers were optimized in succession, and a five-hidden-layer MLP was obtained with an average classification accuracy of up to 90%. Compared with traditional CCA, the MLP achieves a larger measurement range, better measurement accuracy, and is less time-consuming.

## Figures and Tables

**Table 1:** Running time of MLP and CCA.

Algorithm	Running Time (s)			
	1	2	3	4
CCA	19.1259	18.8495	18.6920	18.5678
MLP	0.4371	0.4639	0.4374	0.4456

## Data Availability

Not applicable.
